# Umbilical Scabies

**DOI:** 10.1590/0037-8682-0220-2025

**Published:** 2025-08-18

**Authors:** Qiuping Li, Xiaokang Xu, Zehu Liu, Xiujiao Xia

**Affiliations:** 1 Department of Dermatology, Hangzhou Third People’s Hospital, Hangzhou Third Hospital Affiliated to Zhejiang Chinese Medical University, Hangzhou, China.; 2 Changxing Skin Disease Hospital, Changxing County, Zhejiang, China.

**FIGURE 1:**
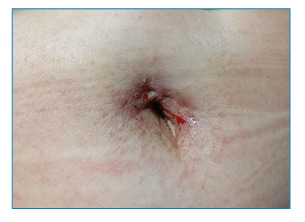
Erythema with bloody scratches located in the umbilical region.

**FIGURE 2:**
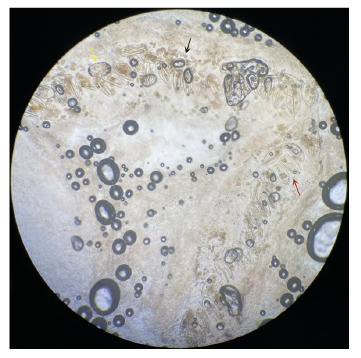
Optical microscopy (x100) of skin scrapings showing abundant eggshell fragments (red arrow), fecal pellets (black arrow), and scattered eggs (yellow arrow) within a mite burrow.

A 59-year-old woman presented to the dermatology clinic with a one-month history of intense itchy rashes in the umbilicus and periumbilical skin. Physical examination revealed erythematous papules with excoriation, crusting, and scaling in the umbilical region (Figure 1). Microscopic examination of skin scrapings collected from the umbilical fossa using a sterile blade revealed abundant *Sarcoptes scabiei* eggshell fragments, fecal pellets, and scattered eggs distributed within a characteristic burrow (Figure 2). The patient was treated with 10% compound sulfur cream applied nightly for five consecutive days, resulting in complete resolution of the lesions.

Scabies is a contagious skin disease caused by the burrowing mite *S. scabiei*. The hallmark skin lesion of scabies is a burrow-like structure, typically located in the interdigital spaces, hands, wrists, axillae, feet, buttocks, and genital regions^1^.

The identification of mites, eggs, eggshell fragments, and mite pellets confirmed the diagnosis of scabies. The life cycle of *S. scabiei* lasts for approximately four to six weeks. During this period, adult females lay two to four eggs per day within burrows in the stratum corneum. Two to four days after laying, the larvae emerge from their shells, leaving behind characteristic eggshell fragments. These remnants often appear as V-shaped, double-leaf fragments because the shell fractures into two connected pieces^2^. Owing to its unique anatomical structure, the umbilical fossa may serve as an optimal incubation site for female scabies mites.
